# Compensatory weight gain due to dopaminergic hypofunction: new evidence and own incidental observations

**DOI:** 10.1186/1743-7075-5-35

**Published:** 2008-12-01

**Authors:** Julia Reinholz, Oliver Skopp, Caterina Breitenstein, Iwo Bohr, Hilke Winterhoff, Stefan Knecht

**Affiliations:** 1 Department of Neurology, University of Muenster, Albert-Schweitzer-Strasse 33, 48129 Muenster, Germany; 2 Department of Pharmacology and Toxicology, University of Muenster, Domagkstrasse 12, 48149 Muenster, Germany

## Abstract

There is increasing evidence for a role of dopamine in the development of obesity. More specifically, dopaminergic hypofunction might lead to (over)compensatory food intake. Overeating and resulting weight gain may be induced by genetic predisposition for lower dopaminergic activity, but might also be a behavioral mechanism of compensating for decreased dopamine signaling after dopaminergic overstimulation, for example after smoking cessation or overconsumption of high palatable food. This hypothesis is in line with our incidental finding of increased weight gain after discontinuation of pharmaceutical dopaminergic overstimulation in rats. These findings support the crucial role of dopaminergic signaling for eating behaviors and offer an explanation for weight-gain after cessation of activities associated with high dopaminergic signaling. They further support the possibility that dopaminergic medication could be used to moderate food intake.

## Background

Eating and dopaminergic signaling are closely related. Food reward and food-reward associated stimuli both elevate dopamine levels in crucial components of the brain reward circuits [1,2]. In fact, food might be the most important natural stimulator of the reward system in the brain [3]. Therefore, overeating may represent an attempt to compensate for hedonic reward deficiency under conditions of reduced dopaminergic activity.

Relative dopaminergic deficiency can be caused by different conditions, for example genetic predisposition or after adaptive downregulation of the dopaminergic system due to preceding overstimulation. Thus, substitutional food intake might explain weight gain after smoking cessation, during antipsychotic medication and in obesity.

A rebound effect of eating behavior after dopaminergic overstimulation could account for the weight gain often associated with smoking cessation, because during smoking, nicotine excites dopamine-containing cells in the ventral tegmental area, resulting in dopamine release in mesolimbic and mesocortical projections [4].

Additionally, an increase in body weight is a side effect of many commonly used drugs. Particularly, antidopaminergically acting neuroleptics, tricyclic antidepressants, lithium, and some anticonvulsants contribute to weight gain. To date, the underlying mechanisms are still poorly understood although interactions with the dopamine system have been implicated [5].

Similarly, in obesity body mass index is negatively correlated with D2 receptor density in the striatum [6,7], which might reflect neuroadaptation secondary to overstimulation with palatable food [8,9]. Thus, increased food intake may be a compensatory behavior for low dopaminergic drive [10]. Stice et al. recently reported that lower striatal activation in response to food intake was associated with obesity. Furthermore, this relation was modulated by genetically determined D2 receptor availability [11].

**Figure 1:**
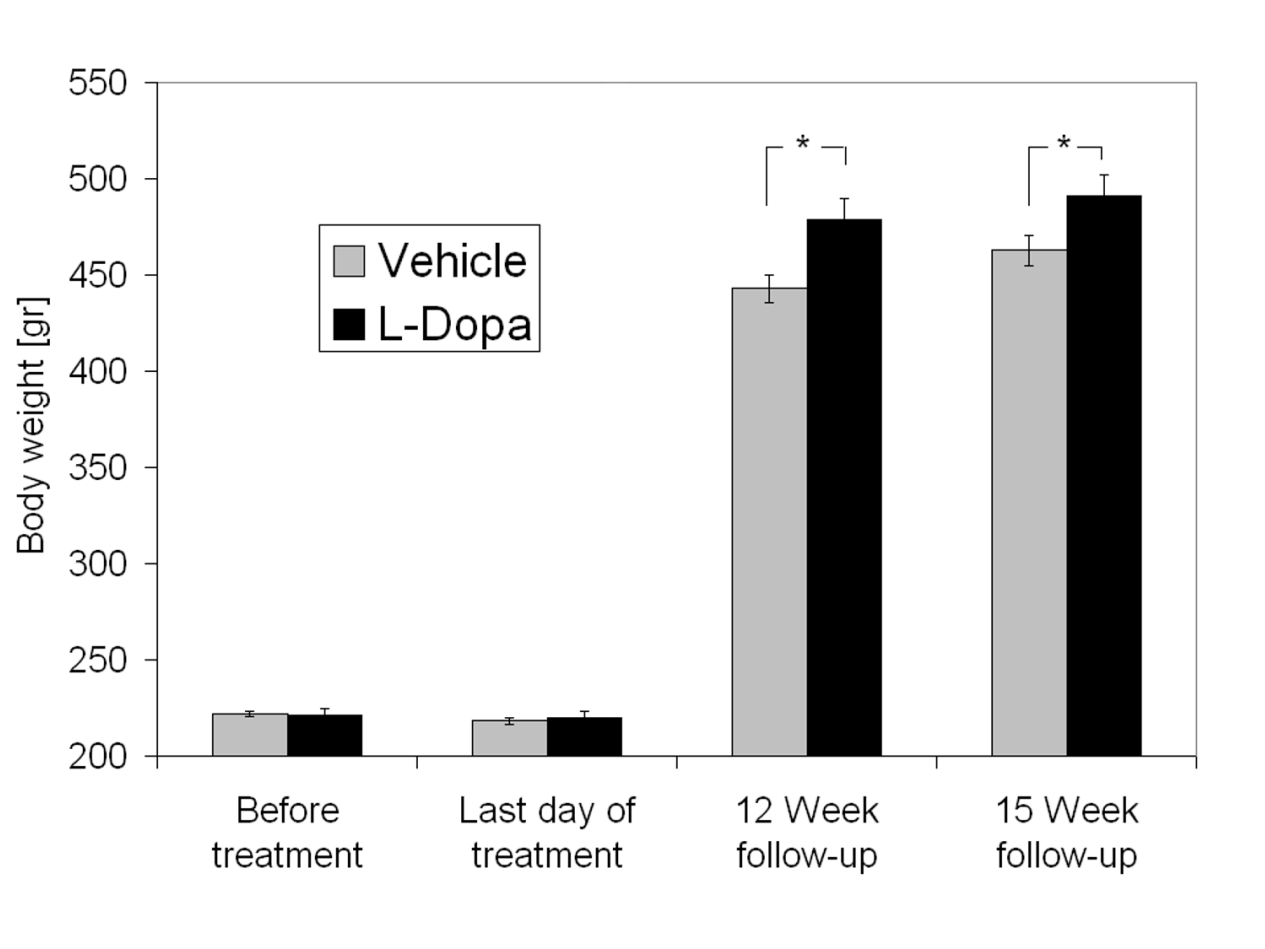
**Effects of L-DOPA treatment on body weight**. Rats treated previously with L-DOPA had the same weight as rats treated with a vehicle solution before and immediately after the treatment, but gained more weight during a follow-up period. Results represent the means ± SD of body weight for each group measured at the respective time point. Asterisk indicates a significant difference (p < 0.05).

**Figure 2:**
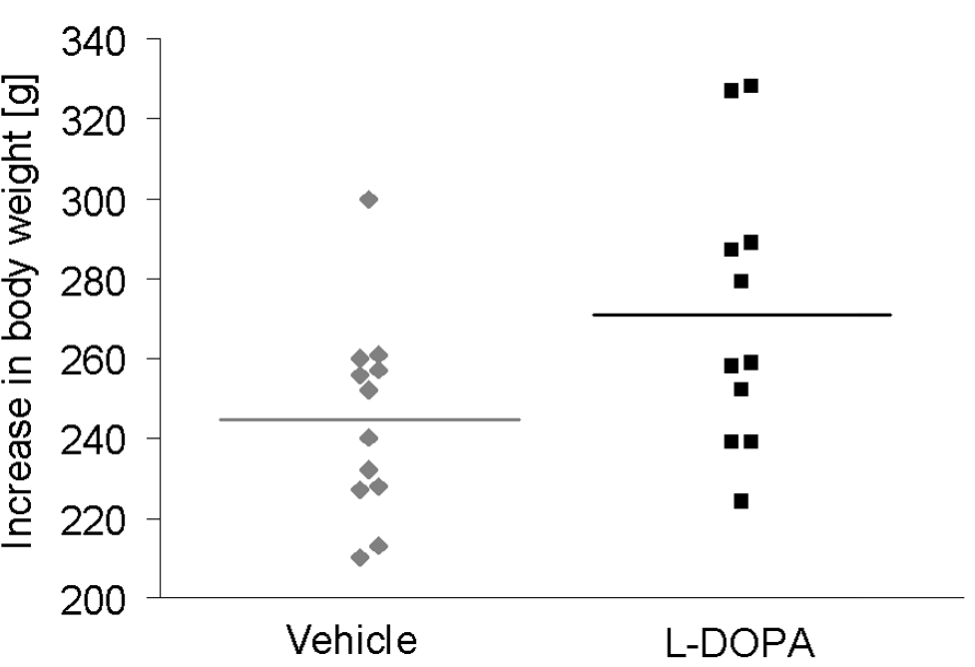
**Individual data on increase in body weight**. All but one rat from the vehicle group gained less weight than the mean weight gain of the L-DOPA group. About half of the rats from the L-DOPA group gained more weigh than almost all rats from the vehicle group. Results represent the individual difference in body weight for each rat from both groups between the last day of treatment and the 15-week follow-up.

These results are in line with our own incidental observation of increased body weight after pharmaceutical dopaminergic overstimulation in an animal model. Regulation of feeding by acute dopaminergic stimulation has already been demonstrated [e.g. [12]], but rebound effects after overstimulation have not been reported. Food restricted rats received the dopamine precursor levodopa over five days and were then withdrawn from dopaminergic medication. Subsequently, animals were allowed to feed ad libitum. Over the next 12 weeks the intervention group gained 15% more weight than the vehicle group (p < 0.01) and continued to be heavier at 16 week follow-up (p < 0.05, see Figure 1 and Figure 2).

## Discussion

There is growing evidence for a role of dopaminergic signaling in the development of obesity. Compensatory eating due to hypofunctionality of the dopaminergic system can not only be based on genetically determined factors, but might also be induced by preceding overstimulation with natural stimulation or pharmacological enhancement. The later was demonstrated by our incidental finding that a decreased dopaminergic tone (relative to a preceding period of extrinsically elevated dopaminergic drive) enhanced weight gain after a period of food deprivation.

While acute administration of levodopa in combination with carbidopa leads to an increase in brain dopamine levels [13,14], diminished dopaminergic responses to external stimulation have been observed after repeated levodopa administration [15-17]. (Over)stimulation of the dopaminergic system by intake of dopaminergic substances or chronic overconsumption of food [10], leads to adaptational processes in the dopaminergic system [18,19]. This downregulation is likely to be complex and seems to involve decreased dopamine synthesis [20], and decreased post-synaptic receptor expression [21,22]. In addition to hedonic or motivational changes in response to food, interactions of the dopaminergic system with adiposity signals might have induced changes in feeding behavior [see [23] for review]. We assume that in our study the hyperdopaminergic state during the repeated levodopa administration induced a hypodopaminergic state after drug discontinuation, which resulted in rebound effects of weight gain as a compensatory mechanism [3].

Dopaminergic modulation of such rebound effects can explain weight-gain after cessation of activities associated with high dopaminergic signaling. Additionally, they offer explanations for individual differences and pharmacological treatment related to post-smoking weight-gain. For instance, in smokers with dopamine receptor polymorphism variants associated with lower dopamine drive, food seems to have greater reinforcing effects as indicated by an increased weight gain after smoking cessation relative to individuals without this variant [24,25].

Our results also raise the possibility that dopaminergic medication may be helpful in preventing compensatory food intake and offer a potential pharmacological treatment of obesity [26]. Increased food reinforcement and weight gain in ex-smokers can be attenuated by bupropion, a dopamine and norepinephrine reuptake inhibitor that raises brain dopamine levels and increases receptor activation [27]. Similarily, after an increase in brain synaptic dopamine via pharmacological inhibition of the dopamine transporter, obese men reduced their energy intake by one third compared to placebo during a meal of highly palatable food [28]. On the other hand dopaminergic treatment in Parkinson's disease or Restless Legs Syndrome may be associated with the inverse effect, i.e. an unwanted weight loss [29].

## Conclusion

Our findings support evidence of dopaminergically induced eating behavior to compensate for low dopaminergic signaling. They should alert us to the possibility that overeating after withdrawal might be a potential side-effect of dopaminergic stimulation. On the other hand, our results also raise the possibility that dopaminergic medication may be helpful in preventing compensatory food intake. These possibilities and limitations of dopaminergic stimulation on motivation merit further investigation.

## Competing interests

The authors declare that they have no competing interests.

## Authors' contributions

JR performed statistical analysis of data, and prepared the final manuscript. OS organized the study and collected the data. BC participated in the conception and design of the main study. IB participated in preparing the final manuscript. HW and SK designed and supervised the main study, SK drafted an initial manuscript.

All authors read and approved the final manuscript.
